# Trans, non-binary and gender diverse adults' experiences of accessing sexual healthcare: a qualitative exploration

**DOI:** 10.3389/fpubh.2026.1871652

**Published:** 2026-10-29

**Authors:** George Burrows, Ingrid Young, Craig Donnachie, Kirstin R. Mitchell

**Affiliations:** 1 School of Health and Wellbeing, University of Glasgow, Glasgow, United Kingdom; 2 Centre for Biomedicine, Self and Society, School of Population Health Sciences, CMVM, University of Edinburgh, Edinburgh, United Kingdom; 3 Department of Psychological Sciences and Health, University of Strathclyde, Glasgow, United Kingdom

**Keywords:** adults, health inequalities, levesque framework, sexual function, transgender (binary and non-binary), United Kingdom (UK)

## Abstract

**Background:**

Trans, non-binary and gender diverse (TNBGD) sexual function has historically been narrowly defined as physical capacities following medical and surgical gender affirming (GA) interventions; and this population's sexual health has been researched more in terms of external influences considered risky rather than being founded in individuals' desires and needs. This can produce discrepancies between services and people who access them. This study explores the service-related barriers, facilitators and compensatory strategies for TNBGD people seeking healthcare related to sexual function.

**Methods:**

Based on qualitative interview data from *n* = 19 TNBGD adults in the UK, the dataset was deductively analyzed using Levesque's framework of healthcare access.

**Results:**

TNBGD people face barriers in distance and transport to appropriate sexual health services, overlaps between sexual healthcare and GA services, and limits in professionals' TNBGD specific knowledge and cultural competence. To navigate these TNGBD people pursued independent knowledge acquisition, were selective during clinical interactions and access, and sought to meet their needs outside clinical settings. The analytical themes demonstrate varied barriers and their interrelatedness. For example, acceptability of a service was informed by the appropriateness of clinician's knowledge and interactions. In turn, TNBGD people's anticipation of un (der) met needs motivated compensatory measures, such as seeking information online or from peers before approaching formal services or sometimes instead of approaching them.

**Conclusions:**

Healthcare services' design and delivery can present significant barriers to TNBGD adults seeking healthcare to support their sexual function. Providers' ways of interacting may offset some of the barriers and TNBGD adults may deploy strategies that enable them to access care that leads to satisfactory health outcomes. However, while significant barriers persist, TNBGD adults are liable to have unmet needs and consequent health inequalities.

## Introduction

Sexual health and wellbeing of TNBGD people are often understood in relation to definitions and frameworks developed with and for cisgender people. Sexual health definitions over the last five decades have variously emphasized physical and mental health, rights and responsibilities, and individuals and populations (1). Sexual function is integral to sexual health, indicated by the reference to absence of dysfunction in the World Health Organization's working definition (2). Conceptualization of sexual health has varied in the extent to which it is considered as primarily biomedical (3), with more recent emphasis on sexual wellbeing either as part of sexual health or, as Mitchell et al. proposed, one of four aspects, along with sexual justice, pleasure and wellbeing, that underpin public health of sexuality (4). Sexual function in trans health research has traditionally been defined exclusively as a physical function (5, 6), with a few more recent studies recognizing subjectivity and social and interpersonal aspects (7, 8). This study elaborates on how social context interacts with individual experiences and perspectives on TNBGD sexual health.

Sexual health is an important health need that is often the focus of what little research exists with TNBGD people; nevertheless, TNBGD sexual health warrants fuller investigation. Sexual function as a gender affirming (GA) treatment outcome is well-established as a clinical research topic (9, 10). Sexual risks and HIV also feature strongly in the literature, including a review indicating high HIV prevalence in trans women (11–13). Trans women are often conflated with men who have sex with men (MSM) (14), obscuring findings on TNBGD sexual health. In policy and guidance, the current (eighth version) international Standards of Care for the Health of Transgender and Gender Diverse People (15) also name sexual health as an important part of GA care. However, a review of UK primary care guidelines for gender minority and trans populations found HIV and GA healthcare most strongly represented, while sexual function and other health priorities were omitted or under-represented (16). A mixed-methods qualitative study found TNBGD people had low expectations of sexual health services meeting their needs (17).

Contextually, UK primary sexual healthcare service delivery spans multiple clinical and non-clinical settings, including general practice, sexual health clinics, third sector (non-profit and charitable) organizations and peer-led organizations. Delivery may be in person or remote, for example online, by telephone or post (18). Healthcare services are delivered by diverse personnel including: medical, nursing or other state regulated healthcare professionals; non-regulated professionals such as development workers or administrators; and those in volunteer roles or peer groups. Healthcare services are fundamental to TNBGD people having the potential to improve and maintain their sexual health. However, healthcare services for TNBGD people, including sexual healthcare, present multiple, complex barriers (19–26), including lack of TNBGD visibility (20, 21, 25), discrimination and stigma (22, 26). The multiple barriers, explored in this study, contextualize the strategies TNBGD people adopt to try to navigate services and fulfill their sexual health needs, hence extending knowledge of this under-researched population and associated complex health challenges.

Levesque's model of healthcare accessibility helps with understand TNBGD people's sexual healthcare service experiences. The model describes access as “the opportunity to have healthcare needs fulfilled” (27) and barriers traverse structural, organizational, interpersonal and individual levels. The model details five dimensions, or qualities, of healthcare service accessibility and five abilities of individuals and populations. Each quality is linked with a corresponding individual ability: the five qualities of services as approachable, acceptable, available, affordable and appropriate; and the related abilities of people to perceive, seek, reach, pay for and engage in healthcare (27–30) (see Figure 1).

**Figure 1:**
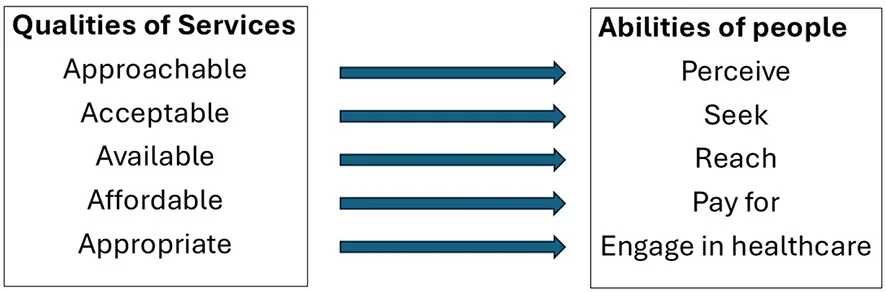
Qualities and abilities in Levesque's model of healthcare access.

Approachability relates to the existence of services and transparency about treatments and services they provide (27). This can include targeted information to raise awareness among one or more subpopulations. The ability to perceive incporates people's knowledge, skills and beliefs about health (27, 30).

Acceptability concerns how systems are organized, administrative processes and the culture and practice of organizations and individual service providers (27, 30, 31). Ability to seek healthcare involves people knowing how as well as having the capacity to find out about and pursue healthcare services (31).

Availability comprises services' accommodation and practical aspects such as geographical location, opening hours and how appointments are booked (27). Specialist sexual health clinics in the UK are predominantly in urban areas and may vary in their targeted service provision for subpopulations; the few TNBGD specific clinics in the UK are located within cities (32, 33). Ability to reach healthcare services relates to the physical attendance at services and is affected by transport, mobility limitations and work or other social constraints (23, 27, 34).

Affordability concerns financial and time resources to meet the requirements set by services (27). UK statutory sexual health services do not have a direct individual financial cost, but may involve greater time commitment to secure appointments and treatments. Private healthcare is less common in the UK than other countries, where health insurance is of greater relevance to affordability (30). Ability to pay relates to people's income generation and expenditure beyond housing, food and other essential living needs (27, 30). The requirement for upfront payments reduces people's ability to pay, even if this is later reimbursed (28).

Appropriateness involves both how services are organized and interpersonal interaction quality relative to the needs of the people requiring the services (27). Services must go beyond adequacy to fit the specific individuals and populations accessing them (27, 34). Ability to engage relates to cognitive and communication skills, knowledge and capacity of people to actively participate in making decisions about their care and following through with treatments (20, 27), although both patient and provider knowledge are necessary for effective TNBGD healthcare (20).

This study aims to better understand TNBGD adults' sexual healthcare access in the UK and addresses the research question: “What do TNBGD adults see as the barriers and facilitators to achieving good sexual function?”

## Methods

### Study design

This study reports on a key finding from a larger, qualitative study that aimed to develop understanding of TNBGD adults' experiences of sexual function, sexual dysfunction and sexual problems (35). The analysis presented here concerns one of three key themes within the study, “navigating the medical sphere” which explored perceived barriers and facilitators to achieving sexual function across all healthcare settings. This article applies Levesque's (27) framework to specifically focus on primary care related experiences, which was the most strongly represented setting identified during the primary analysis.

Purposive sampling was undertaken in non-clinical settings, with an initial recruitment target of up to 30 participants to gather rich data to underpin strong analysis. The inclusion criteria were: age 18 years and over; resident in the UK; identifying in some way as trans (with prospective participants invited to take a broad interpretation); and having some experience of sexual activity (alone or with others). We aimed for variation in key attributes (ages; GA treatment experiences; feelings of gender dysphoria; socio-economic status; ethnicity; spiritual or philosophical perspective; general health; and disability) but did not formally screen for these during recruitment and no quotas were set. The first author disseminated the call for participation primarily through social media (mainly Twitter (now X), also by third parties in some Facebook groups where group administrators permitted this), supplemented with planned and opportunistic in person interactions. The call was amplified through snowballing by participants and other direct contacts, such as colleagues, acquaintances and staff in third sector organizations.

From early on, participation enquiries came predominantly from younger people, similar to other TNBGD sex and relationships studies (36, 37). During recruitment, additional steps were taken to increase the sample heterogeneity, with targeted communication for underrepresented UK regions and genders. The first author used social media messages expressly inviting trans feminine people to consider being interviewed with the aim of increasing trans feminine participant recruitment. This resulted in an additional participant who was assigned female at birth and who identified as trans and feminine.

Consent was confirmed via a two-part paper form, with sections for completion before and after the interview; this meant that participants knew what they had discussed before deciding whether to agree to the possibility of data reuse. Participant information forms were publicly available during the recruitment period, and a consent form was sent in advance of interviews so that participants could consider their decision. Following full ethical approval from the University of Glasgow's College of Social Science ethics committee, recruitment occurred between July 2019 and March 2020.

The research team consisted of a lead researcher with support from three additional researchers. Team members have various gender and sexual perspectives from a white, predominantly UK context. Our approach is founded on prioritizing and respecting TNBGD people's identities, bodily autonomy and self-determination, alongside the right to health.

### Data collection and analysis

Each participant was interviewed in person by GB. A topic guide supported the semi-structured approach. Topics explored included:

- Meaning of the term “sexual activity”
- Meaning of “sexual relationship”
- Influences on sexual life
- Wanting or getting support around sex
- Effects of medicine on TNBGD people's sexuality and sex life.

All interviews used primarily verbal communication and were audio recorded on an encrypted digital device and transcribed. Verbal communication is less compatible with some people's access needs (38, 39); visual methods can increase accessibility (39). Printed terms on cards supported consistent presentation of sexual (dys) function related terms. GB and one participant agreed a reasonable adjustment; planned questions in line with the topic guide were presented in text format alongside spoken delivery to aid communication and accessibility of research participation.

Interviews were conducted in participants' homes or university premises to optimize participants' privacy and comfort to engage, along with an appropriately quiet environment for audio recording and therefore data collection quality. All audio recordings were transcribed by a professional transcription company with whom the research team agreed a secure data sharing protocol that was General Data Protection Regulation compatible and satisfied the additional sensitivity requirements relating to this small population and highly sensitive topic. In all cases, GB checked transcript quality and accuracy, which aided data familiarization.

The data were analyzed using “Framework Analysis”, a five stage thematic analysis that enables both inductive and deductive approaches (40–42). The five analytical stages undertaken in accordance with Framework Analysis were: “data familiarization”; “identifying a thematic framework”; “indexing”; “charting”; and “mapping and interpretation”. Through discussion between all authors, five main themes were generated which align with Levesque's framework and support understanding of barriers, facilitators and strategies for TNBGD people navigating the medical sphere.

## Results

### Participant characteristics

Nineteen TNBGD adults located in England and Scotland were interviewed. Contacting organizations in Northern Ireland garnered no enquiries and one enquiry from Wales in response to social media messages did not lead to participant recruitment. Participants were aged from 18 years to mid-60s (specific ages are not reported to reduce the potential for deductive disclosure of participants' identities) and were mostly trans masculine (*n* = 15), non-monogamous (*n* = 11), had no GA genital surgery (*n* = 16), and had current or past disabilities or health conditions (*n* = 17). One participant had genital surgery during adulthood for medical reasons unrelated to gender affirmation. Table 1 shows pseudonymised participant demographics and GA treatment experiences.

**Table 1:** Participant demographics and gender affirming treatment experiences.

Name	Gender	Sexual orientation	Monogamous?	Age group (years)	Urban, rural or other location	Gender affirmation			
						Medical	Surgical	Social	Other
Pip	Agender; non- Binary	Queer	Mostly monogamous	18–25	Urban	No	No	Not stated	Not stated
Jim	Agender	Not stated	Polyamorous	18–25	Urban	Testosterone	No	Not stated	Not stated
Stephen	Male, with transsexual history	Bi, pansexual, attracted to both gender or all genders	Monogamous	26–35	Urban	Testosterone	Chest surgery	Yes	Not stated
Luke	Trans man, mostly, maybe	Bisexual, queer	Non-monogamous	26–35	Urban	Testosterone	Chest surgery	Yes	Not stated
Vic	Non-binary; “I don't use the label “trans” very often”	Bi, pan or queer	Non-monogamous	36–50	Urban	No	No	Not stated	Not stated
Lee	Non-binary	Exclusively dated women	Monogamous	26–35	Urban	Testosterone	Top surgery	Not stated	Not stated
Rita	Non-binary, assigned female at birth	Queer, polysexual	No	18–25	Urban	No	No	Not stated	Considering future treatment
Helen	Trans fem	Don't really know	Not stated	18–25	Rural	Past HRT	No	Not stated	
Barry	Agender; non-binary; trans masculine	Pansexual, queer	Not stated	18–25	Urban	Not stated	Not stated	Not stated	Awaiting gender clinic
Paul	Man (possibly with a trans history)	Bisexual (not excluding non-binary people)	Not stated	26–35	Suburban	Testosterone	Top surgery	Yes	Not stated
Kim	Non-binary and trans	Bisexual or pansexual; occasional greysexual or sex repulsed	Yes	36–50	Urban	No	No	Not stated	Intra-uterine device
Christie	Trans man	Gay man	Not stated	Over 50	Urban	Testosterone	Unknown	Yes	Not stated
Ollie	Non-binary	Attracted to women; LGBT (preferred term)	Yes	26–35	Urban	No	No	Yes	Not stated
Simone	Female; transgender	Lesbian	Not stated	36–50	Urban	Female hormones; testosterone suppression	Vaginoplasty	Yes	Not stated
Rich	Non-binary; trans man; man (if having to choose either man or woman)	Gay or queer	No	26–35	Urban	Testosterone	Chest surgery; phalloplasty	Yes	Not stated
Noah	Trans male (to cis people); 95% male, 5% neutral; some days it's whatever	Queer; mostly attracted to more masculine	No	18–25	Urban	Testosterone	Chest surgery	Yes	Not stated
Jenny	Trans woman	Attracted to femininity	Yes	Over 50	Not stated	Estrogen	Vaginoplasty	Yes	Vasectomy
Frankie	Non-binary, probably gender fluid	Pansexual	Yes	26–35	Urban	No	No	Not stated	Chest binder
Davey	Trans woman; non-binary; gender fluid; expressional fluidity; sometimes don't really feel a connection to gender at all	Bisexual; pansexual	Yes	18–25	Urban	No	No	Gender fluid / expressionally fluid	Medical circumcision (in adulthood)

### Self-defining sexual healthcare needs

Having a healthcare need is foundational in understanding the process of accessing healthcare (27). Before reporting findings on the themes of services' qualities and people's abilities, the highly subjective nature and varied ways of understanding sexual function and associated healthcare needs must be acknowledged. The same experience can be viewed very differently between individuals, raising the question of what constitutes a healthcare need.

“Just because something that isn't a problem for me, even though I‘ve experienced these things, doesn't mean that they're not something that would really worry and concern and upset somebody else going through them.” (Kim, non-binary and trans, 36–50 years).

The meaning of *having* a healthcare need is important. In this analysis, subjective definitions are prioritized over externally defined biomedical ones if there is a discrepancy between the two.

The findings that follow are structured around the five abilities of people and corresponding qualities of services, as presented by Levesque (27) and introduced above. Example quotes for each theme are presented in Table 2.

**Table 2:** Themes and example quotes.

Theme	Example quotes
1. Discerning the mode of healthcare according to perception of service vs. sexual health need	“when I was getting sexually tested for STIs, I would go to the doctors and just get a DIY kit so they wouldn't get the nurse to do it for me, I'd just go into the toilet, take the swab myself and give it back to the reception for them to send away for testing, which was a really nice way of doing it without the stress of someone else getting all up inside you.” (Rita, non-binary, assigned female at birth, 18–25 years) “I'm with a great GP at the moment, and she's great, and I feel like she's on my side… I had a bit of an alarming symptom. And she was, not worried, but she took, I think, all the precautions, and she put referrals through and made them, marked them as urgent. […] But had a nice, sort of understanding tone, and not judgmental, or anything. So yeah, I see that as, I saw her as respecting the whole person that I am, and that includes any sexual, just my life, any part of my life, or decisions that I make...” (Stephen, male with transsexual history, 26–35 years) […T]he [Practice] Nurses took really tremendous care of me scar-wise and everything. They did all the after-care for that [phalloplasty], so that kind of restored my faith in the practice as a whole but I still don't feel comfortable…like the fact that it [gynecological pain] impacted my life so hugely and they kept sending me away going, no, no, that's fine, and that actually was a huge impact on my sex life as well...[The TNBGD sexual health clinic] were, like, okay, we believe you, which is something I don't generally get told by medical professionals anyway, so that was quite a powerful moment there.” (Rich, non-binary, trans man, man (if having to choose either man or woman), 26–35 years) “I'm not sure if I imagined it, but I felt like she [the nurse with the title “Sister”] might have like laughed a little bit, not in a mean way, but almost that she found it kind of funny that someone was struggling that much. And like I said, I don't know if that's because, in my head I was thinking, she's like a [religious] sister person, obviously she could probably guess, like given my gender expression and stuff, that I was probably queer. Even I'm not sure if I actually told her that I was…then when I went back to the reception, to sort of reschedule it, I do recall saying, if possible can you make sure it's not the same person, it's somebody else? So, yeah, eventually it was rescheduled, they got somebody else, and it was so much easier. Like, not that it was pain free or anything, but it was not as awful as that time.” (Ollie, non-binary, 26–35 years)
2. Finding alternative sources of information and support	“I've been looking around for, are there certain techniques that might help, are there certain toys that might help, are there certain ways I can get that sensation back, or anything like that? And there's just no resources, or even people or a group or forum or something, to kind of talk to about it. It feels silly saying it, but I kind of felt alone.[…] I've just had to figure that out myself. There's nothing that explained how vibration and using a vibrator on the scar tissue affects it…” (Davey, trans woman, non-binary, gender fluid, expressional fluidity, sometimes don't really feel a connection to gender at all, 18–25 years) “I know the [specific LGBTQ+ organization], they run workshops and things, sometimes. I went to one, sex was like a small part of it, but we had other discussions as well. So yeah, there's groups like that, and workshops like that, that are quite helpful. Otherwise, it would just be friends…” (Stephen, male with transsexual history, 26–35 years) “Well, having kink friendly friends, who don't mind talking about it, is very valuable. And I find Facebook groups, and places like that, quite good, because there is this ability to be slightly less self-conscious, and the ability to know that you've expressed yourself accurately. Because you can re-read, you can clarify, you can do all the things that I‘ve found difficult, verbally. And so that's pretty good. But definitely, medical services, like I've said, I‘ve had to hide my gender from psychologists…” (Vic, non-binary, “I don't use the label ‘trans' very often”, 36–50 years)
3. Proximity to sexual healthcare services	“You know, I struck lucky because I went to a menopause clinic where the doctor does not just double but treble duty. The doctor isn't just the menopause doctor, she is also general gynae and also gender identity. So that is triple whammy, that's the kind of doctor that they should have everywhere.” (Kim, non-binary and trans, 35–60 years) “they've been really wonderful about going outside their remit because they knew that there was nobody else that was helping me. I think [the trans sexual health clinic] is the only place I've gone to that every time I've been, like, oh, right, I've got this problem and then they've actually come back with more information and helped me work through it and I haven't been the one going please read this and then help me.” (Rich, Non-binary, trans man, man (if having to choose either man or woman), 26–35 years) “I've had a few, but just gone for tests, mostly I just get them posted and send them back again” (Noah, trans male (to cis people), 95% male 5% neutral, some days it's whatever, 18–25 years)
4. Paying the price when state healthcare falters	“So, I went private for my testosterone in my 1 year of uni, because I had a part time job, actually, from the 1 week of freshers I started then, and I'd got on to T [testosterone] by July because I'd saved up enough money. And then, I spent the next year and a bit saving up for top surgery, and I might get an appointment for GIC next month, but it's a bit late now, so what can they do.” (Noah, trans male (to cis people), 95% male 5% neutral, some days it's whatever, 18–25 years) “So, when I was kind of having my little flurry, a few years back, I kind of was a regular […]And then he [the sexual health doctor] told me about the politics of PrEP not being available in England [on the NHS], and then he said, I'm the doctor on the local branch of this clinical trial, and if you want to, you can sign up to it, and you can get PrEP for free then basically. And I was like, oh okay, he said all you have to do is come back every 3 months. And I was like, okay, that sounds good.” (Paul, man (possibly with a trans history), 26–35 years)
5. Self-determination and willingness to engage in different settings	“Yes, so like, yes, my GP is like honestly, they're terrible to talk about, because they just assume like whoever I'm with is cis, and then when I went to a GP recently to have an STI test, like a few weeks ago, they were asking about my sexual partners. I was like, oh, I've got a partner who is non-binary, and they just did not have a clue what to do with that information, they were just like, what does that even mean? Like are they a girl or a boy? I was like they're neither. Then they had to ask like what sort of genitalia they had, which had nothing to do with me having an STI test, because like I only had to pee in a cup, like we didn't need to talk about my partner's genitals, but that's what happened.” (Jim, agender, 18–25 years) “[M]y GP and I don't really talk about it [sexual health]. I kind of prefer it that way.[…] I think I was getting cysts that were bursting and possibly having my ovaries twist, but everybody refused to diagnose me or work with me, including this GP, who said, oh, it was just IBS.[…] So I've never really been comfortable with her as a trans person since then.” (Rich, non-binary, trans man, man (if having to choose either man or woman), 26–35 years) “Well, when I was...it's very, actually really problematic, like when I first went to the gender clinic for my first kind of initial appointment, they seemed to be really like correlating like sex acts to like gender and asking me really strange and really invasive questions about that.” (Lee, non-binary, 26–35 years)

### Discerning the mode of healthcare according to perception of service vs. sexual health need

Most participants' accounts conveyed confidence in recognizing whether their own sexual experiences constituted a healthcare need, relying primarily on subjective definition. Where participants expressed doubt, their definitions drew more heavily on formal definitions and external knowledge sources, including medicine, media and peers, to determine whether they had a healthcare need. If or when a healthcare need was perceived, the extent of services' approachability then became relevant.

Participants discussed a wide range of services. For instance, doctors, including General Practitioners (GPs), were a commonly mentioned provider type. Nurses were the other main type of practitioner providing direct care, with administrative staff also influencing participants' experiences of in person clinical services. Service types spanned general population, LGBTQ+ and young people's clinics delivered by statutory (National Health Service) organizations; third sector organizations, including specialist sexual health clinics for TNBGD people and their partners; remote services centered around online, telephone and postal communications.

Some in person and remote testing services discussed included self-sampling in which individuals, rather than healthcare professionals, used swabs to collect samples to test for ‘sexually acquired infections' (43, 44). For example, Rita's GP surgery supported self-sampling. (See also Table 2).

“when I was getting sexually tested for STIs, I would go to the doctors and just get a DIY kit so they wouldn't get the nurse to do it for me, I'd just go into the toilet, take the swab myself and give it back to the reception for them to send away for testing, which was a really nice way of doing it without the stress of someone else getting all up inside you.” (Rita, non-binary, assigned female at birth, 18–25 years).

Both the nature of healthcare staff interactions and participants' perceptions of the service overall contributed to service approachability. Approachability, therefore, lay within participants' perception of the service, including its staff.

Some locations may restrict or not offer minimally interactive services, or individuals may need to see someone, such as to discuss symptoms. When participants had sexual health needs for which they perceived the need to see a healthcare practitioner, they strongly desired choice over which specific practitioners to engage with or avoid. Stephen described interactions with GPs as “a very mixed bag” and this view was fairly consistent across participants, in contrast to much more positive interactions with nursing staff (Table 2).

Nursing staff, when encountered, were often found to be supportive and approachable; their care and overall approach contributed to satisfaction with interactions. For example, Rich's primary care (practice) nurses supported his sexual health through their skill and professionalism in their wound care after his phalloplasty surgery. However, some participants recalled unsatisfactory interactions with nurses. For example, Ollie (see Table 2) was equivocal about the nurse's professional role due to the title “sister” on her name badge, which they interpreted as being associated with religion based on past experiences. Ollie explained during the interview about not wishing any kind of vaginal penetration, therefore finding vaginal examinations stressful. Ollie's account of the clinical examination by the nurse demonstrates how their (di)stress interacted with the nurse's perceived inappropriate response. This prompted Ollie's subsequent avoidance of that practitioner and increased caution in using that healthcare service. Despite Ollie's caution, they were still willing to engage with the service and used a strategy of carefully negotiating ways to do so, including requesting a different practitioner.

Ollie's experience contrasted with others' much more positive experiences, such as Paul's series of interactions with a sexual health doctor (see Theme 4), in which the affective qualities of the care interaction were foundational to accessibility of care in practical terms. Combined, the service's multiple qualities gave Paul the opportunity to have his healthcare needs met, ultimately improving his sexual health.

### Finding alternative sources of information and support

Participants tended to have the skills and resources for learning about sexual health topics and services that they deemed relevant to their needs. Abundant information was reported as being available via the internet, such as sexual community discussion forums, and was favored over other sources, owing partly to greater anonymity (45). Most participants gravitated toward the internet to find information to understand or improve their sexual health, although Davey's information search was unsuccessful; having not received sufficient or relevant information at the time of their surgical treatment (adult circumcision), they found self-exploration as the only remaining option.

“I've been looking around for, are there certain techniques that might help, are there certain toys that might help, are there certain ways I can get that sensation back, or anything like that? And there's just no resources, or even people or a group or forum or something, to kind of talk to about it. It feels silly saying it, but I kind of felt alone.[…] I've just had to figure that out myself. There's nothing that explained how vibration and using a vibrator on the scar tissue affects it…” (Davey, trans woman, non-binary, gender fluid, expressional fluidity, sometimes don't really feel a connection to gender at all, 18–25 years).

Others, like Vic and Stephen (Table 2), communicated with peers in person, online or both and asked them for information about sexual experiences, discerning whether these constituted a healthcare need, and identifying healthcare service availability and acceptability.

Statutory services aimed at general (cisgender) populations were less acceptable and encountered more through circumstance or lack of realistic alternatives. For example, Davey (above) reported minimal information about sexual implications of adult circumcision. Services focussing on TNBGD people, or LGBTQ+ people more generally, were the most acceptable to participants. For instance, Stephen (Table 2) discussed how an LGBTQ+ organization had many “helpful” options. When acceptable formal services were unavailable, most participants, including Stephen and Vic (Table 2), turned instead to friends and peers for knowledge.

### Proximity to sexual healthcare services

UK Sexual health clinics are mainly in urban locations. Aside from GPs, participants did not discuss experiences of rural sexual health services. Participants who were able to reach urban areas benefitted from the availability of sexual healthcare services that met their needs particularly as TNBGD people. For example, Kim lived near a menopause clinic that delivered TNBGD-aware gynecological healthcare.

“You know, I struck lucky because I went to a menopause clinic where the doctor does not just double but treble duty. The doctor isn't just the menopause doctor, she is also general gynae and also gender identity. So that is triple whammy, that's the kind of doctor that they should have everywhere.” (Kim, non-binary and trans, 36–50 years).

Rich had the financial and practical means to travel to one of the few specialist UK TNBGD sexual health services that went “outside their remit”, positively surprising Rich at not having to “[be] the one going please read this and then help me”. Other target populations are sometimes prioritized for remote sexual healthcare options, for example testing kits by post for young adults (18), as mentioned by Noah (Table 2). Additional influences on ability to reach healthcare include mobility related disability, which Kim reported. Had Kim not happened to live near an appropriate service, their mobility related access needs may have been unmet, indicating the impact of multiple marginalization.

### Paying the price when state healthcare falters

GA care, which impacts sexual health, is underserved within the NHS; gender clinic waiting times can be prohibitive, which led Noah to pursue work to pay privately for more timely GA healthcare, leading in turn to better health, including sexual health.

“So, I went private for my testosterone in my 1 year of uni, because I had a part time job, actually, from the 1 week of freshers I started then, and I'd got on to T [testosterone] by July because I'd saved up enough money. And then, I spent the next year and a bit saving up for top surgery, and I might get an appointment for GIC next month, but it's a bit late now, so what can they do.” (Noah, trans male (to cis people), 95% male 5% neutral, some days it's whatever, 18–25 years).

Political and ethical factors affect whether treatments are state funded and provided through the NHS and, therefore, care is affordabile. Pre-Exposure Prophylaxis (PrEP) is medication to prevent an HIV negative person becoming HIV positive. At the time of data collection, participants accessing PrEP reported that it was available only through clinical trials. This relates indirectly to affordability because trials are contingent on funding. Two participants, including Paul (see Table 2), discussed accessing PrEP. Without NHS sexual healthcare access, Paul would not have known about or had free access to PrEP.

### Self-determination and willingness to engage across settings

Overwhelmingly, participants had sufficient knowledge to make health related decisions; the main constraint was willingness to engage with services according to its appropriateness. Rich, for example, was reluctant to engage following prior experience of a particular GP (Table 2). According to Rich, his GP blocked accurate assessment, referral, diagnosis and treatment of severe pain that Rich deduced was not Irritable Bowel Syndrome but was ovarian in origin and highly detrimental to his sexual health. A different service later facilitated referral and found it to be necessary, demonstrating that the GP's refusal resulted in lack of appropriate healthcare. This omission irreparably diminished Rich's trust and contributed to him disengaging with that GP about his sexual health. Jim reported a more straightforward situation of seeking testing without onward referral and compensated for the GP's incorrect assumption that “whoever I'm with is cis” by educating the GP about the meaning of having a non-binary partner and how that related to sexual activity.

“Yes, so like, yes, my GP is like honestly, they're terrible to talk about, because they just assume like whoever I'm with is cis, and then when I went to a GP recently to have an STI test, like a few weeks ago, they were asking about my sexual partners. I was like, oh, I've got a partner who is non-binary, and they just did not have a clue what to do with that information, they were just like, what does that even mean? Like are they a girl or a boy? I was like they're neither. Then they had to ask like what sort of genitalia they had, which had nothing to do with me having an STI test, because like I only had to pee in a cup, like we didn't need to talk about my partner's genitals, but that's what happened.” (Jim, agender, 18–25 years).

Many UK GA healthcare clinics are accommodated in buildings specializing in sexual healthcare. Participants overwhelmingly discussed crossover of sexual health with other health services, especially GA care, as inappropriate and reducing their willingness to fully engage. Participants mostly sought to keep these two aspects of their health separate, using selective disclosure only of what they deemed relevant to the given service they were attending (for example, Lee, Table 2). A key concern was that overlaps of staffing or location of gender and sexual healthcare services could lead to transfer of information disclosed in sexual healthcare settings and result in being held back in progressing with GA care. Most participants who mentioned gender and sexuality together were also wary of their inappropriate conflation. Atypically, Kim endorsed overlapping of sexual and GA health services, indicating it positively contributed to meeting their sexual healthcare needs (see theme 3).

Clear separation of gender and sexual healthcare services elicited greater confidence for participants to discuss all aspects of health they considered relevant to their sexual health. Typically, participants recounted more positive, appropriate experiences through clinics specializing in sexual health and no perceived crossover of staffing or through co-location with GA healthcare. The services discussed most positively, indicating high appropriateness and willingness to engage, were services focussed on a specific subpopulation, such as MSM (as in Paul's experience) or TNBGD populations for those able to reach these. Staff in specialized TNBGD sexual health services separate from the NHS were willing to prioritize the patient's presenting sexual health needs (for example Rich, Table 2). This circumvented barriers within statutory services, whether these barriers were ingrained into the system, related to interpersonal barriers from practitioners or individual barriers in reaching and using services effectively.

Practitioners' proactive seeking of information was highly valued by Rich and other participants. Based partly on experiences, such as Jim's, above, participants generally expected to have to undertake a lot of intellectual effort as a compensatory strategy to overcome practitioners' lack of TNBGD-specific knowledge.

## Discussion

Barriers and facilitators to TNBGD people's healthcare access manifest in many ways, both in healthcare services and in those who may have a healthcare need. With reference to Levesque's model, each of the five dimensions and abilities and associated barriers and facilitators were evident in this study of TNBGD adults. Our study found that self-determination was key for TNBGD people understanding their sexual health and addressing their associated care needs. Financial status, geography and mobility influenced the range of healthcare opportunities available. Perceptions of TNBGD people's sexual healthcare needs are complex and varied among TNBGD individuals and sexual healthcare providers. The nature of available services also varied geographically, with greater urban availability. Individuals used compensatory strategies, most notably selective disclosure in sexual health services that were perceived as overlapping with GA healthcare. Practitioner development opportunities and service delivery approaches can facilitate TNBGD primary sexual healthcare access and reduce associated inequalities.

### Self-determination and social influences on sexual health and access to care

Participants' non-perception of sexual healthcare need may be a barrier to accessing care, or, as for some participants, a health need exists only if an experience or phenomenon is perceived as a health concern. This perception can come from external cues, such as how experiences are categorized and whether a corresponding health service exists. The domain of medicine is, therefore, one of the social influences on TNBGD people's perceptions of their sexual health needs. Opportunities to pursue healthcare were also shaped by individual social factors, such as: proximity to services; transport, mobility constraints and time availability; social connections, such as peer groups, to discover sexual health information and the existence and reputation of services.

Financial status and employment both interact with transport affordability and free time to travel to services. Primary healthcare, including sexual health testing and treatment, is free to all in the UK regardless of immigration status (46). However, other indirect costs of accessing and reaching healthcare can be constraining, and may prevent people from seeking care (30). For TNBGD people, travel affordability barriers can prevent use of otherwise accessible sexual healthcare service. Income and cash flow limitations or the indirect barrier of being unable to take time off paid work can make traveling to one of the few specialist TNBGD sexual health clinics unfeasible. The absence or under-availability of GA treatments and care can be detrimental to TNBGD people's sexual health. Starting or increasing paid employment to fund healthcare is not universally possible. The ever-tighter constraints on state funded GA treatments and diminishing support within general practice make affordability of GA treatment an increasingly important influence on TNBGD sexual health.

Lack of privacy and being asked to account for one's movements can impinge on the opportunity to access sexual healthcare, which has been noted as a barrier among younger populations living with parents (47). People who live alone or do not require such privacy may therefore find it easier to have the opportunity to reach sexual healthcare services. Disabled people can find that services are not available to them in contrast to non-mobility impaired peers (34), as identified in this study.

Similar to chronically ill populations (31), TNBGD people's needs are systemically excluded, necessitating involvement in healthcare systems and patient-centered approaches to reduce barriers to healthcare (48). This study emphasizes the importance of self-determination in TNBGD people defining their own sexual health problems and associated care needs and the constraints on individuals' opportunities to act on their self-defined needs, similar to others' emphasis on the importance of knowledge derived from TNBGD people's own lives (49). Furthermore, if institutions and practitioners within them clearly demonstrate their cultural competence (27, 50, 51), knowledge and willingness to engage with TNBGD populations, TNBGD people are more willing to approach and engage with them. Similar to US research with trans masculine youth (52), this study identified a preference for seeking information online or from peers or partners prior to considering accessing staffed services. Greater distance from culturally competent sexual health services can also incentivise marginalized patient groups seeking sexual health information online, as two North American studies found (53, 54).

### Influences of practitioners and health services on accessibility of TNBGD sexual healthcare

Sexual function is an under-recognized health need. Furthermore, a mismatch between needs of TNBGD people as a marginalized population vs. the nature of available services can render services inaccessible (31). This is related to “cultural competence”, which concerns providers' knowledge and skills in TNBGD sexual healthcare and is fundamental to services being accessible (25, 47–49). There is no single cultural norm or need among TNBGD populations, and the small population size and social hostility toward transness and gender divergence (55–57) reduce the likelihood of services being delivered by TNBGD staff. Discriminatory healthcare interactions, stigmatizing behaviors and lack of practitioner knowledge are associated with poorer sexual health outcomes in TNBGD people (48, 58).

Incorrect assumptions by practitioners was a further set of barriers to sexual healthcare access identified in this study. For example, primary healthcare practitioners incorrectly assumed patients' sexual activities, their partners' bodily features and associated risks, constituting an inappropriate clinical interaction. This erroneous approach corresponds with other studies of TNBGD sexual healthcare (59) as well as reflecting cis/heteronormativity embedded in sexual health research that does not allow TNBGD experiences to be appropriately reflected (60, 61). Inappropriate questions around TNBGD status and misattribution of symptoms to being TNBGD are two ways in which practitioners' interactions may be inappropriate and therefore are access barriers (62, 63). Imposing a cisgender category onto gynecological health conditions, such as endometriosis, also produces systemic, policy and interpersonal (practitioner) level barriers for TNBGD populations and individuals (26).

Even anecdotal information about others' negative experiences could undermine potential acceptability, and participants in this study drew on peers' knowledge to discern the acceptability of services. However, formal development opportunities for healthcare professionals may be difficult to find, with *ad hoc* approaches leaving practitioners to rely on colleagues organizing knowledge sharing, and with knowledge inconsistently available in all services and locations (24). Medical educators also sometimes avoid discussing politicized health issues, including sexual and reproductive health (64), suggesting that professional attitudes could contribute to knowledge gaps in TNBGD health as a highly politicized minority population.

Peers were participants' other main source of information about the availability of services. Peers also shared experiences that then shaped participants' impressions and progression to approach services (20). In a review of PrEP access among bisexual (cisgender) men (47), closer affiliation with LGBT communities was associated with higher awareness of PrEP and lack of representation in health promotion information was a barrier to knowing about and using PrEP. Lack of clear TNBGD representation can also be a barrier to TNBGD sexual healthcare, for example presenting cervical screening as being for women or conflating trans women with cisgender gay and bisexual men (25, 65). When TNBGD representation is lacking in services' own publicity and health promotion materials, peer connections and anecdotal information may be more readily trusted by TNBGD people.

Willingness to use available sexual healthcare services was a stronger barrier in this study than the ability to seek. Participants were concerned that available services would not sufficiently meet their needs (66), which demotivated participants from seeking access to services at all or by limiting the content of their future interactions.

#### Overlapping service location and staffing produce conflicting priorities for TNBGD people's sexual and other healthcare needs

UK gender clinics are sometimes delivered in buildings specializing in sexual healthcare, for example Lothian and Glasgow (67, 68). Ostensibly, co-location is more convenient, while also creating complexity and sometimes barriers to sexual healthcare access. However, participants were predominantly concerned that co-location could lead to sexual healthcare information being divulged to gender clinicians who might stifle their progress with GA treatments. In agreement with this study, a Scottish needs assessment found that TNBGD patients may withhold or selectively disclose information to guard against delays or withholding of GA treatments (69). When GA treatment provision is situated in a building primarily for sexual health services, the sexual health care is liable to be less trusted by TNBGD people who are accessing or hoping to access GA care. Co-location of the two services can reduce “acceptability”, therefore can constitute a significant barrier to sexual healthcare access. However, some evidence from a TNBGD sexual health clinic that also offers GA care services suggests a high level of acceptability (70), suggesting that the barrier is not from the co-location itself, but from a discrepancy between gender clinicians' and GA care patients' perspectives on whether sexual experiences and interests interact with gender and, if so, their relative importance in GA care decision-making. TNBGD people who perceive little or no relevance might experience this discrepancy as a misattribution of gender divergence to sexuality, akin to “trans broken arm syndrome” (62, 63), in which sexual interests and experiences, rather than gender dysphoria or incongruence, are externally perceived as evoking a desire for GA treatments.

#### Timing and availability of appointments limiting opportunities for accessing sexual healthcare

Appointment availability can be a difficulty in primary healthcare. General Practice in UK, for example, is renowned for the “8am scramble” in which patients are expected to phone for a same day appointment (71). Those who fail to get through quickly enough or cannot call at that time will miss out. Furthermore, TNBGD people are often highly discerning about who they are willing to discuss sexual health needs with, which is at odds with the approach of patients being allocated whoever happens to be covering the duty that day, therefore the consultation, even if secured, may not be suitable for the person's presenting need.

In sexual healthcare, reduced availability of sexual healthcare appointments can be a barrier to accessing healthcare. Availability constraints may be caused by non-attendance of those who have secured an appointment (72) and have been attributed to sustained cuts to funding of statutory sexual health services (24). However, in this study, delays were discussed in connection to GA treatment services rather than services primarily focussing on sexual health.

### Anticipatory knowledge acquisition as a self-advocacy strategy in the face of epistemic injustice

Engagement in healthcare services requires knowledge, communication, decision-making involvement and following through with any agreed care or treatments (27, 31). Owing to typically low expectations of practitioners having the relevant knowledge and cultural competence (21, 24, 48, 73), this study found TNBGD people relied on their own knowledge acquisition in readiness to self-advocate and educate healthcare practitioners during consultations, increasing likelihood that their healthcare needs would be met. The routineness of this anticipatory knowledge acquisition iwas highlighted by the exceptional instances of healthcare practitioners having independently sought further information and used this in subsequent clinical interactions.

Gaps in understanding and communication between practitioners and patients can be particularly problematic with health issues that are not easily observed or measured (74). Practitioners are also in positions of power, in which they may perpetuate or disrupt stigmatization of TNBGD patients (22). Alongside the dynamic of practitioner as “gatekeeper” who ultimately holds the decision-making power over whether to offer care, treatment and referral (48, 75), differences in understanding and communication can be understood with reference to epistemic injustice (76). This includes “testimonial injustice” in which first hand experiences are disregarded or considered less credible than accepted authorities, such as clinicians' judgment and research based evidence (77, 78), and “hermeneutical injustice” in which marginalized perspectives are less represented in the knowledge that society overall draws upon and therefore are less readily understood (76). Involvement in healthcare depends on having or developing sufficient shared understanding between practitioners and patients. In this respect, practitioners' TNBGD cultural competence is important for providing shared understanding, which helps to remedy the epistemic injustice TNBGD people face and therefore supports TNBGD people's ability to engage in healthcare interactions.

GPs' unwillingness to accept participants' own accounts of their symptoms and needs can be understood through the framework of epistemic injustice. GPs' lack of understanding of TNBGD participants' sexual practices and relational context created communication gaps. Attempts to engage with GPs about symptoms, such as gynaecolgical pain, were sometines insufficient to bring about the necessary healthcare, diminishing willingness to engage with those practitioners while remaining willing and able to engage with other, receptive services and practitioners. This study found that participants tended to be highly knowledgeable about their health. Unlike in other research (73), lack of individuals' knowledge, was not the barrier to engagement. Interestingly, another study noted that TNBGD participants' knowledge levels exceeded their own estimations of their understanding of a sexual health issue (21).

## Study strengths and limitations

This study is founded on a rich dataset derived from high quality interaction during face to face interviews between the first author and participants. In contrast with many previous studies that recruited via specialist GA healthcare services (79), this study engaged participants discussing broad experiences, including those who had not sought GA healthcare services, of whom some did not intend to. The inclusion of three participants (16%) aged over 50 is an unusual and positive aspect of this study relative to many studies that mainly or exclusively recruit younger adults. Where other studies may have selected participants with binary gender identities who are sexually attracted to binary gender identified partners (80), this study engaged many non-binary participants and sexual experiences with non-binary partners. Proactive enquiry about participants' needs for disability related adjustments facilitated the engagement of disabled participants, including those with communication and mobility impairments. This study adds a UK perspective which is very underrepresented in the existing evidence base and a qualitative approach in contrast to the more abundant quantitative studies.

Data collection for this study was undertaken immediately prior to the inception of the COVID-19 pandemic, since when organization of healthcare services have changed. The socio-political context in the UK and internationally has also become more hostile toward TNBGD people, which could influence TNBGD people, healthcare professionals and the systems within which the practice in ways not represented in the data. The non-representative sampling method mean these findings are not generalisable. Nevertheless, future comparison with other studies could aid identification of similarities and differences.

## Implications of this research

Barriers and facilitators may not always be clearcut and universal. For example, on a systemic level, perceived or actual overlap in services may present as a barrier or facilitator in TNBGD sexual healthcare access and optimum engagement. On the one hand, colocation of sexual health and GA services can give the impression of the potential for seepage of information (typically from sexual health to GA services), so that TNBGD people attending both services may fear that full discussion of sexual experiences and needs may prejudice their progress with GA care. On the other hand, overlap of staffing across GA and reproductive health services may lead to delivery of better care owing to the practitioner's TNBGD awareness and integration of this knowledge when delivering reproductive healthcare. A more balanced mix of separate and overlapping sexual healthcare and GA healthcare should be made available, combined with transparency to patients about overlaps in staffing, record keeping or other routes through which patient data may be shared across these services. This will increase trust in general and especially if crossover is shown not to be detrimental to GA healthcare progress.

This study indicates distance, or ability to travel, can be pivotal for TNBGD people accessing services that deliver the required care (70). The findings of this study indicate gaps in healthcare professionals' knowledge. This supports the need for greater training, which was identified as a research priority in a TNBGD Delphi study (81). Considered alongside the benefits of specialist services, this supports the need for TNBGD culturally competent practitioners and specialist services that TNBGD people can feasibly travel to.

Individuals' compensatory strategies have varying degrees of efficacy and reliability and involve differing levels of effort. The efficacy of information seeking as a strategy may be constrained by an individual's access to information and critical thinking skills to discern the credibility of sources. To alleviate the increased burden on TNBGD populations, healthcare services should facilitate staff development and specialist knowledge acquisition, proactively provide for TNBGD people's sexual health needs in their service delivery and be transparent about the extent to which they do so.

## Conclusions

The primary sexual healthcare services accessed by study participants were varied. However, the variety and quality of services did not comprehensively satisfy the range of participants' needs and potentially those of other TNBGD individuals with similar sexual experiences, health needs, abilities and opportunities for accessing primary sexual healthcare services. Individual, interpersonal and systemic barriers and facilitators to TNBGD adults navigating the medical sphere ranged from straightforward to more sophisticated and were intelligible within Levesque's framework. Insights into individuals' compensatory strategies rest on the foundation of the barriers and facilitators that this theoretical framework helped the authors to elicit.

When barriers seem too great, perhaps even when attempting to use compensatory strategies such as selective disclosure or paying for private treatments, TNBGD people seek sexual health information outside formal sexual healthcare services to try to learn how to self-manage or resolve issues. There remains, too, the possibility of TNBGD people avoiding sexual health issues and care services altogether, reinforcing previous research (24, 82). Consequently, this population remains under-served by many healthcare services and liable to exclusion from care and, therefore, the full potential to optimize their sexual health.

Beyond the strategies used by TNBGD individuals, healthcare professionals can reduce barriers, for example by combining a supportive attitude with independent pursuit of knowledge of TNB sexual experiences and related healthcare needs. In addition to self-led professional learning, structured training could support more practitioners to increase their knowledge and cultural competence. In light of escalating hostility, UK doctors have urged medical practitioners, healthcare organizations and professional bodies to take urgent action to counter the barriers and resulting discrimination against TNBGD patients (83). Nevertheless, individual patients and practitioners are unlikely to singlehandedly remove cultural and systemic barriers, such as constrained resources for professional development or colleagues not accepting a referral to their specialism.

## Data Availability

The datasets presented in this article are not readily available because the highly sensitive topic, potential for deductive disclosure and lack of consent from all participants make this dataset unsuitable for routine sharing and reuse. Requests to access the datasets should be directed to George Burrows George.Burrows@glasgow.ac.uk.
